# Supplementary material for: Different effects of reward value and saliency during bumblebee visual search for multiple rewarding targets
Source: Anim Cogn. 2021 Jan 30;24(4):803–14. doi: 10.1007/s10071-021-01479-3 (PMC8238720; doi:10.1007/s10071-021-01479-3)
Supplement: Supplementary file 1 — Supplementary file1 (PDF 118 KB) [file 10071_2021_1479_MOESM1_ESM.pdf]

Electronic Supplementary Material

Article title: Reward Value Is More Important Than Physical Saliency During Bumblebee Visual Search For Multiple Rewarding Targets

Authors: Vivek Nityananda and Lars Chittka

Corresponding author: Vivek Nityananda

Behavioural Sciences and Psychology, Biosciences Institute, Newcastle University, Henry Wellcome Building, Framlington Place, Newcastle Upon Tyne, NE2 4HH, UK.

email: vivek.nityananda@newcastle.ac.uk

Journal Title: Animal Cognition

Table S1: Distance in hexagonal units between the different colours used in the experiments, as well as betweeen the colours and the background used in the experiments.

	Blue	Cream	Red	Yellow	Fuchsia	Green Background
**Blue**	0.00	0.13	0.42	0.41	0.31	0.45
**Cream**	0.13	0.00	0.43	0.38	0.35	0.47
**Red**	0.42	0.43	0.00	0.14	0.15	0.04
**Yellow**	0.41	0.38	0.14	0.00	0.25	0.18
**Fuchsia**	0.31	0.35	0.15	0.25	0.00	0.16
